# The Amsterdam Wrist Rules to reduce the need for radiography after a suspected distal radius fracture: an implementation study

**DOI:** 10.1007/s00068-019-01194-2

**Published:** 2019-09-20

**Authors:** Marjolein A. M. Mulders, Monique M. J. Walenkamp, Nico L. Sosef, Frank Ouwehand, Romuald van Velde, Carel Goslings, Niels W. L. Schep

**Affiliations:** 1 Trauma Unit, Department of Surgery, Amsterdam UMC, Location Academic Medical Center, University of Amsterdam, P.O. Box 22660, 1100 DD Amsterdam, The Netherlands; 2 Department of Surgery, Spaarne Gasthuis, P.O. Box 770, 2130 AT Hoofddorp, The Netherlands; 3 Emergency Department, Amsterdam UMC, Location Academic Medical Center, University of Amsterdam, P.O. Box 22660, 1100 DD Amsterdam, The Netherlands; 4 Department of Surgery, Tergooi Hospitals, P.O. Box 10016, 1201 DA Hilversum, The Netherlands; 5 Department of Surgery, Onze Lieve Vrouwe Gasthuis, P.O. Box 95500, 1090 HM Amsterdam, The Netherlands; 6 Department of Trauma and Hand Surgery, Maasstad Hospital, P.O. Box 9100, 3007 AC Rotterdam, The Netherlands

**Keywords:** Distal radius, Fracture, Trauma, Decision rule, Radiograph, Implementation

## Abstract

**Purpose:**

While most patients with wrist trauma are routinely referred for radiography, around 50% of these radiographs show no fracture. To avoid unnecessary radiographs, the Amsterdam Wrist Rules (AWR) have previously been developed and validated. The aim of the current study was to evaluate the effect of the implementation of the AWR at the Emergency Department (ED).

**Methods:**

In a before-and-after comparative prospective cohort study, all consecutive adult patients with acute wrist trauma presenting at the ED of four hospitals were included. Primary outcome was the number of wrist radiographs before and after implementation of the AWR. Secondary outcomes were the number of clinically relevant missed fractures, the overall length of stay in the ED, physician compliance regarding the AWR, and patient satisfaction and experience with the care received at the ED.

**Results:**

A total of 402 patients were included. The absolute reduction in wrist radiographs after implementation was 15% (*p* < 0.001). One clinically irrelevant fracture was missed. Non-fracture patients without wrist radiography due to the AWR spent 34 min less time in the ED compared with non-fracture patients who had a wrist radiograph (*p* = 0.015). The physicians adhered to the AWR in 36% of patients. Of all patients who did not receive a radiographic examination of the wrist, 87% were satisfied.

**Conclusion:**

Implementation of the AWR safely reduces the amount of wrist radiographs in selected patients and consequently reducing the length of stay in the ED.

**Electronic supplementary material:**

The online version of this article (10.1007/s00068-019-01194-2) contains supplementary material, which is available to authorized users.

## Introduction

In most hospitals, patients with wrist trauma are routinely referred for radiographic examination. However, many of the radiographs are negative. For example, no fractures of the wrist were found in 58%, 47% and 75% of all such radiographs by Van den Brand et al., Walenkamp et al., and Karaca et al., respectively [1–3].

Currently, no clinical guidelines exist to endorse decision making regarding this radiograph referral. This may result in unnecessary radiographs and increased time spent at the ED, increased workload, and additional healthcare costs [4–7]. A thorough history and physical examination is important and may provide guidance in the decision to request a radiograph. The value of physical examination findings as predictors for wrist fractures has previously been studied [7–9]. However, these studies were limited by small study populations and did not propose a clinical decision rule. Encouraged by the supposed overuse of radiological resources due to the lack of clinical guidelines, a clinical decision rule in adults has been developed and validated: the Amsterdam Wrist Rules (AWR) [3]. Based on age and a number of clinical variables, the AWR calculates the probability of a fracture of the distal radius in patients suspected of having a distal radius fracture. The recommendation whether or not to obtain a radiograph of the wrist is based on this probability [3]. The AWR has been externally validated and has shown a reduction in radiographs obtained of 14.2%, and a sensitivity and specificity for detecting fractures of the distal radius in adults of 98% (95% CI 97–100%) and 25% (95% CI 19–31%), respectively.

The next step is to determine if the rule can be successfully implemented in regular clinical practice [10, 11]. Therefore, the aim of the current study was to evaluate the effect of the implementation of the AWR at the ED.

## Methods

### Study design and population

This implementation study was designed as a before-and-after comparative prospective cohort study. A cohort of patients in which the AWR was implemented (after group), was compared with a historical reference group in which the AWR has been developed and validated (before group) [3]. To diminish patient variation and obtain comparable cohorts, patients in the before-and-after groups were included in the same four hospitals. Approval was obtained from the medical ethics review committee on October 8th 2014, without the need for informed consent. This study is registered with the Dutch Trial Register (NTR5074).

All consecutive adult patients presenting with acute wrist trauma at the ED of one academic and three teaching hospitals were included. Acute trauma of the wrist was defined as any energetic accident involving the wrist, such as a fall on outstretched hand within 72 h preceding presentation at the ED. Patients who sustained multiple injuries with an Injury Severity Score of greater than 15, patients whose radiographs were requested prior to their consultation at the ED (e.g. by their general practitioner), patients who sustained a wrist fracture in the past 3 months or patients in whom the injury occurred more than 72 h prior to the presentation at the ED, were excluded. A log of patients who were screened for eligibility was kept for each participating centre.

**Fig. 1:**
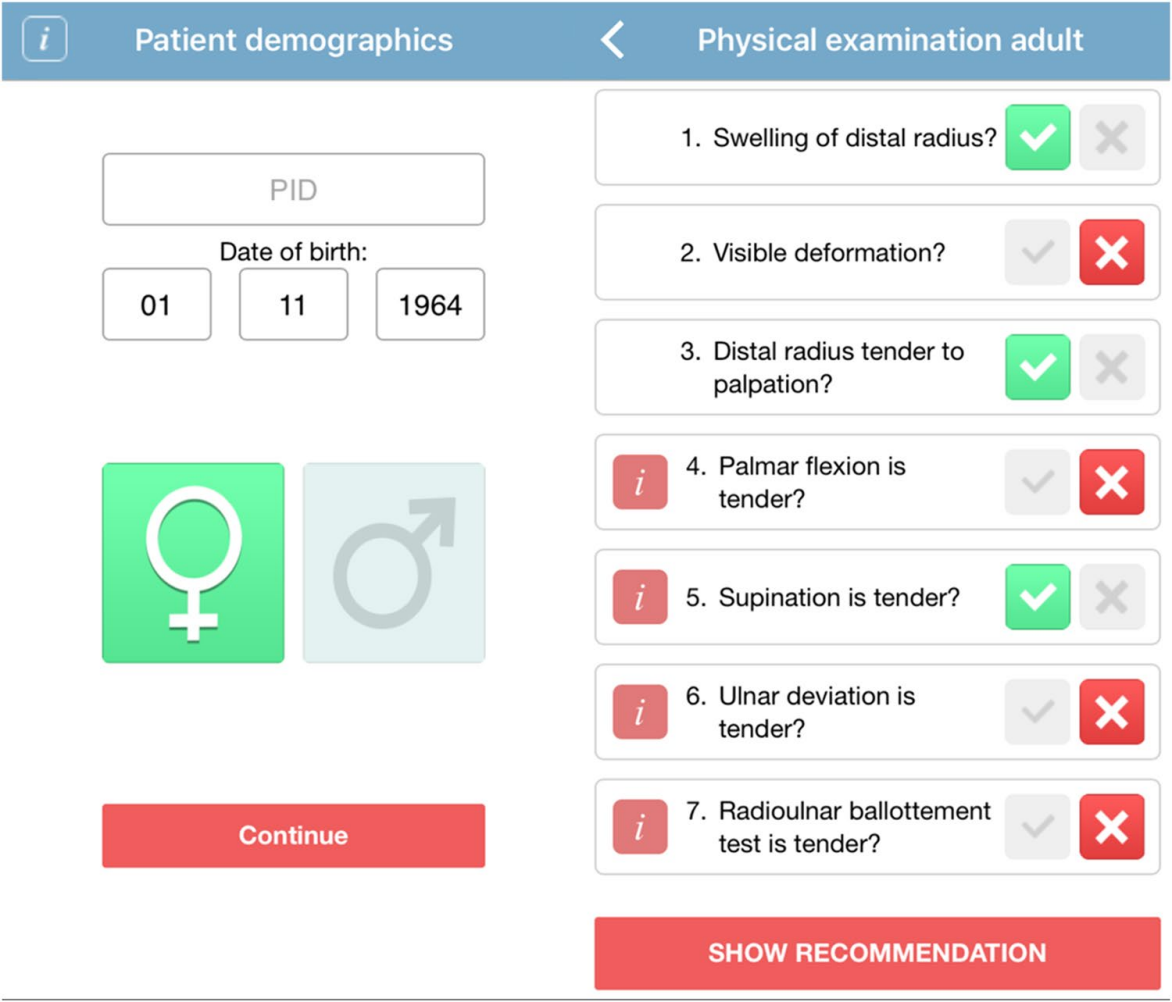
AWR mobile application: patient demographics and clinical findings

**Fig. 2:**
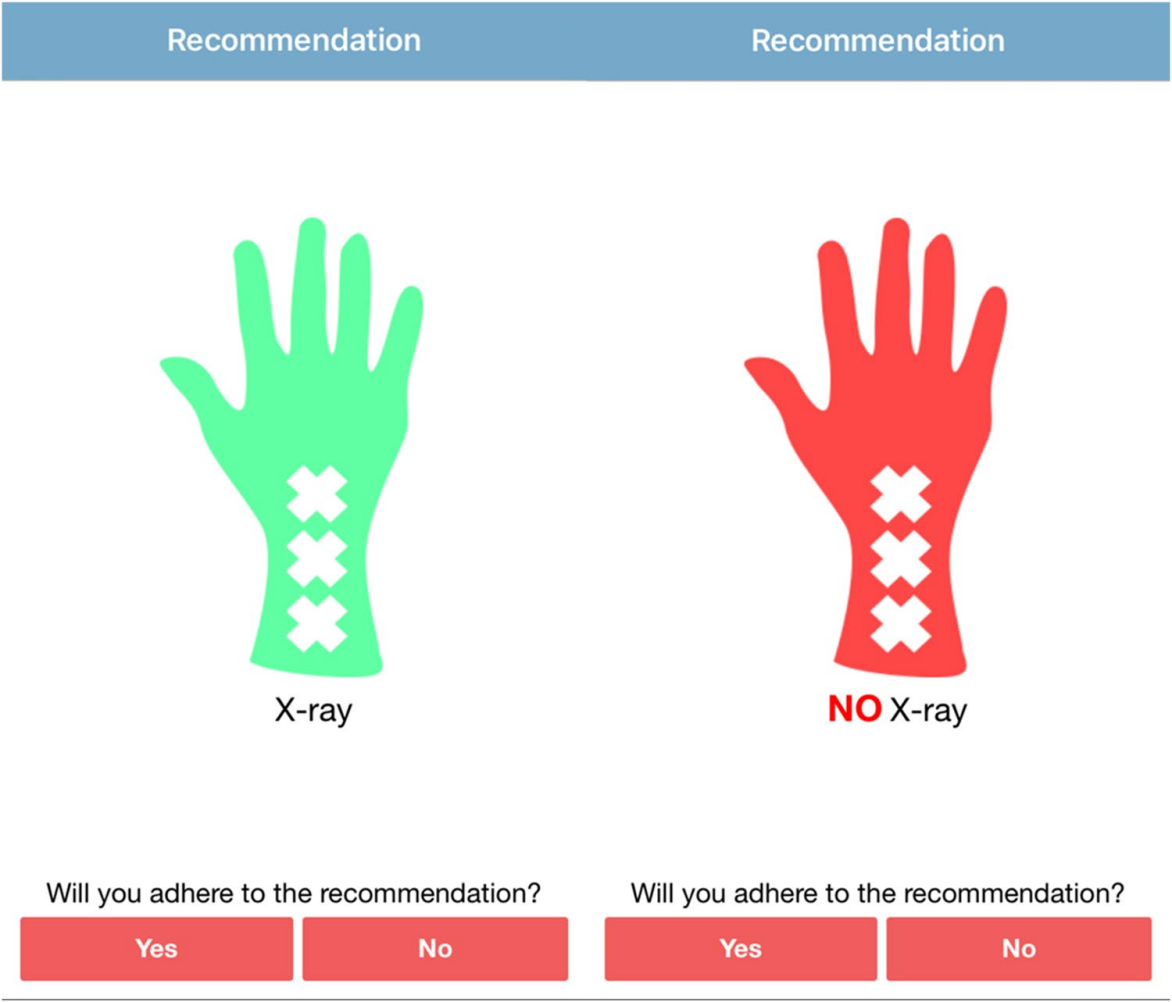
AWR mobile application: recommendation to make a wrist radiograph or not

A smart phone application was developed for use of the AWR: the Amsterdam Wrist Rules application. Patients were entered into the study using this smart phone application. Patient characteristics, including date of birth and sex, and clinical findings during physical examination, were entered into the application (Fig. 1, Online Resource 1). Based on these findings, the AWR algorithm calculates the chance of having a fracture of the distal radius and gives a recommendation on whether to obtain a radiograph of the wrist (Fig. 2). The AWR application was also available on the study website (www.amsterdamwristrules.nl). The anonymous data were safely restored at a secure server, only accessible by the coordinating researcher.

## Outcomes

The primary outcome was the difference in the number of wrist radiographs before and after implementation of the AWR. A wrist radiograph was defined as a posterior–anterior (PA) and lateral radiograph of the distal radius and the carpal bones. Secondary outcomes were the numbers of clinically relevant missed distal radius fractures, the overall length of stay in the ED before and after implementation of the AWR, physician compliance regarding the AWR, and patient satisfaction and experience with the care received at the ED.

A distal radius fracture was defined as the presence or disruption of one or more of the cortices of the distal radius [12]. A fissure and small avulsions of bony fragments were considered to be fractures as well. We defined a clinically relevant missed fracture as a fracture for which prognosis or treatment, including treatment with plaster, closed reduction and an operative treatment, would have been affected by a delayed or missed radiographic diagnosis.

If no radiograph was acquired, patients were contacted after 7–10 days by phone. Patients were invited to visit the outpatient clinic if the patient failed to meet all of the following criteria: (1) pain has decreased, (2) ability to use wrist has improved, (3) able to lift more than 2 kg, (4) ability to push open a heavy door, (5) has returned to normal daily activities excluding sports, and (6) no plans to see a physician about wrist. At this point, referral for any additional workup was at the discretion of the treating physician. In addition to these questions, patients were asked if they consulted another physician related to the trauma of the wrist and if this physician acquired a radiograph of the wrist and gave additional treatment.

Overall length of stay in the ED was defined as the time patients entered the ED and the time patients left the ED. Difference in length of stay in the ED was defined by comparing non-fracture patients without a wrist radiography due to the AWR with non-fracture patients who had a wrist radiograph.

**Fig. 3:**
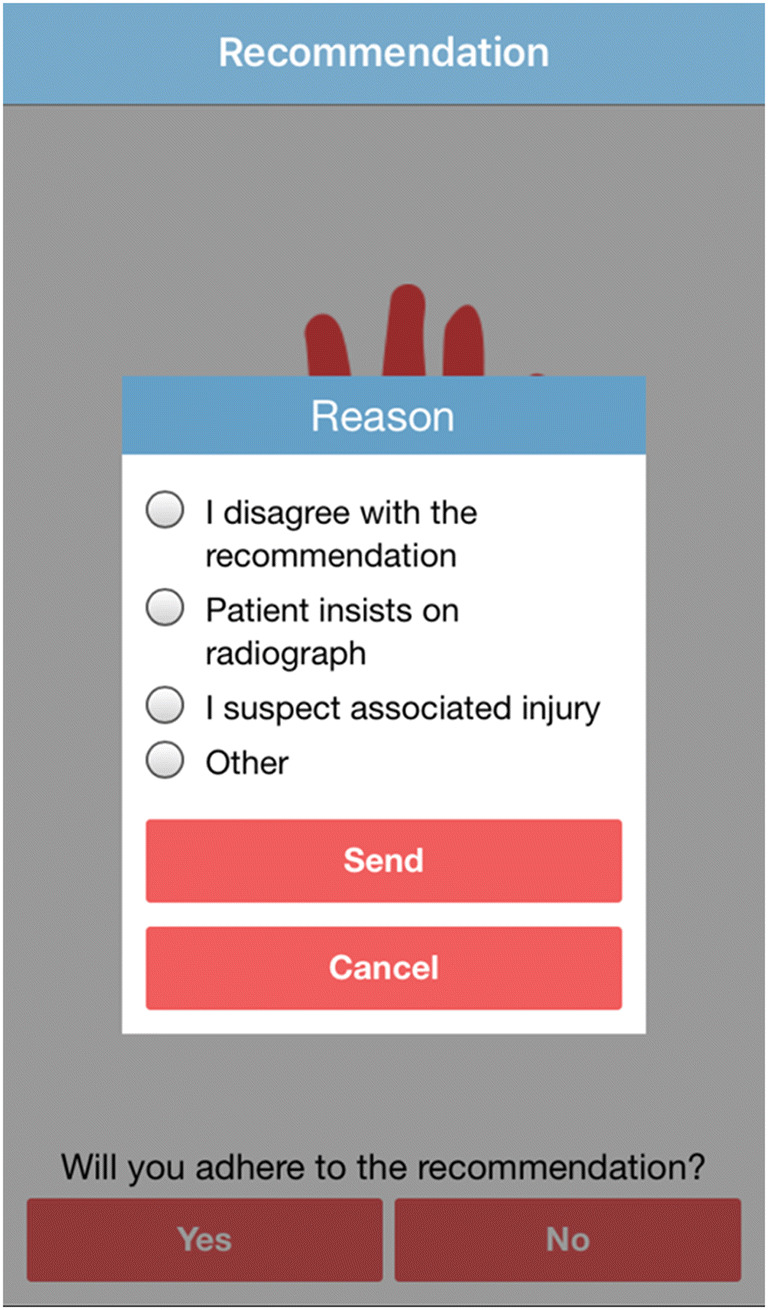
AWR mobile application: adherence of the physicians

Physician compliance regarding the AWR was assessed in the smart phone application, with an additional question after the recommendation was given. If the physicians indicated that they were not planning to adhere to the recommendation, four possible answers could be given: (1) I disagree with the recommendation, (2) patient insists on radiograph, (3) I have the suspicion of an associated injury, and (4) other (Fig. 3).

For patients in whom no radiograph was acquired, patient satisfaction and experience with the care received during their consultation at the ED was assessed during a short telephone survey after 1 week. Patients were asked if they were satisfied or not and if they felt secure without a radiograph of the wrist having been obtained. If not satisfied, they were asked if they would have felt more secure if a radiograph of the wrist would have been made. Additionally, they were asked if they would have been willing to wait longer at the ED to be 100% sure that a distal radius fracture was ruled out.

### Sample size and statistical analysis

The sample size calculation was based on the primary outcome: the difference in the number of wrist radiographs. We assumed 90% of all patients with a suspected distal radius fracture were sent for radiography. For the sample size calculation, we considered a minimal reduction of wrist radiographs of 9% to be feasible. Consequently, with an alpha of 5% and power of 90% and using the standard formula for superiority trials, this resulted in 342 patients per group. Presuming a loss to follow up of 10%, inclusion of at least 377 patients with wrist trauma in whom the AWR were applied was required. The same number of patients was required for the historical reference group.

General descriptive statistics for both groups on patient characteristics at baseline were performed including factors such as sex, age and fracture characteristics. Differences in sex and fracture characteristics between both groups were compared using a Chi square test, and the difference in age was compared using a Mann–Whitney *U* test. The same applied for differences in patient and fracture characteristics between the included cohort of patients and the missed inclusions. The primary outcome, the proportion of patients referred for radiography before and after implementation, was compared using a Chi square test. Secondary outcomes were analysed using either an independent *T* test or a Mann–Whitney *U* test for continuous data and a Chi square test for categorical data.

## Results

### Characteristics of study subjects

**Fig. 4:**
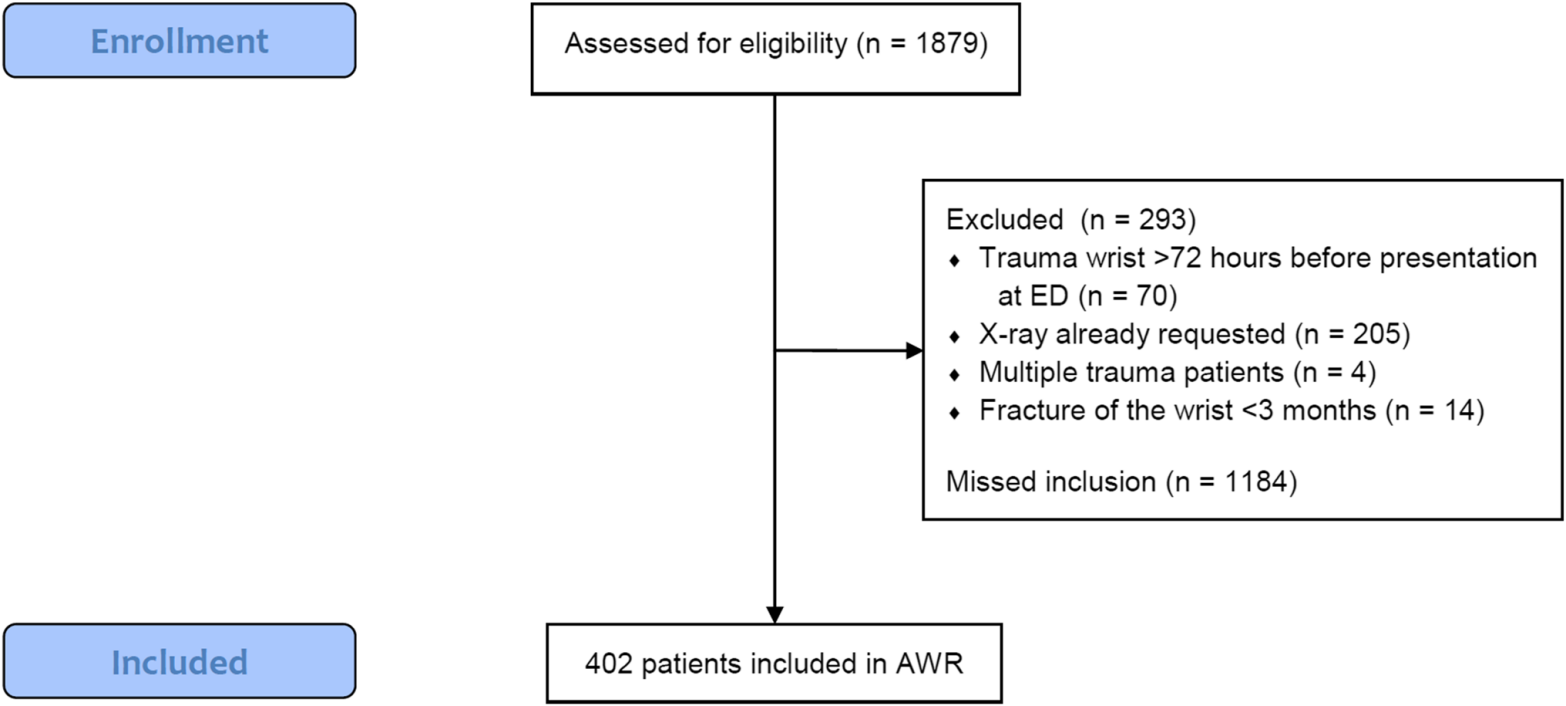
Flow diagram of patient selection. Missed inclusions are patients who fulfilled the inclusion criteria but were not included by unknown reasons

From November 2014 to January 2016, 402 adult patients were included, of which 35% were at the academic hospital and 65% at the teaching hospitals. Since the registration of non-included patients in the teaching hospitals was incomplete, a chart review was performed to verify the number of excluded patients and missed inclusions in these three hospitals within the study period. A total of 1879 patients were eligible for inclusion of whom 293 patients were excluded and 1184 inclusions were missed (Fig. 4). The cohort of missed inclusions had a significantly lower distal radius fracture percentage and the patients were significantly younger compared to the cohort of included patients (Online Resource 2).

The median age of the included patients in the after group was 51 years (IQR 32–67 years), and 61% of the patients were female. Of all included patients, 44% sustained a fracture of the distal radius. The historical reference (before) group consisted of a cohort of 854 patients, included between November 2010 and June 2014. The patient and fracture characteristics between both groups were comparable (Table 1).

**Table 1:** Baseline characteristics before and after implementation of the AWR

	Before implementation AWR, *N* = 859	After implementation AWR, *N* = 402	*P* value
Age (median (IQR))	50 (31–63)	51 (32–67)	0.294
Female (%)	60.5	60.7	0.957
Distal radius fractures (%)	43	44	0.814
Extra-articular (%) Intra-articular (%)	38 62	46 54	0.071

*N* number, *IQR* interquartile range

### Reduction in radiographs and clinically relevant missed fractures

The absolute reduction in wrist radiographs was 15% (99% versus 84%; *p* < 0.001). Before implementation of the AWR, no radiograph was requested for 1% of patients, compared to 16% after implementation (Table 2). One fracture was missed following the recommendation of the AWR. This 55-year-old woman was intoxicated during her ED visit. The patient was contacted after 1 week by phone and she indicated that symptoms were still present. Therefore, the patient was invited to the outpatient clinic. A radiograph showed an extra-articular fissure for which the patient was treated with a removable splint for 4 weeks. This fracture was considered clinically irrelevant. No other patients received an additional radiograph of the wrist or additional treatment elsewhere.

**Table 2:** Primary and secondary outcomes

	Before implementation AWR, *N* = 859	After implementation AWR, *N* = 402	*P* value
Wrist radiographs (%)	99.4	84.1	< 0.001*
ED length of stay (h) [median (IQR)]	1:59 (1:25–3:05)	2:12 (1:31–3:13)	0.074

*IQR* interquartile range

*Statistical significance

After implementation, the AWR correctly identified all 176 clinically relevant distal radius fractures and thereby achieving a 100% sensitivity and negative predictive value (Table 3).

**Table 3:** Performance of the Amsterdam Wrist Rules after implementation among 402 patients with wrist trauma

	No distal radius fracture	Distal radius fracture
AWR recommends radiograph	162	176
AWR recommends no radiograph	64	0
Sensitivity (% [95% CI])	100% [97.3–100%)	
Specificity (% [95% CI])	28.3% [22.6–34.7%]	

*CI* confidence interval

Performance was tested based on the 15.3% reduction in wrist radiographs, applying the definition of a clinically relevant distal radius fracture

### ED length of stay

There was no significant difference in length of stay in the ED before and after implementation of the AWR (Table 2). However, compared with non-fracture patients who had a wrist radiograph after implementation of the AWR, those discharged without a wrist radiograph spent significantly less time in the ED (118 min versus 84 min; *p* = 0.015) (Table 4).

**Table 4:** Characteristics of 225 non-fracture patients with and without a radiograph of the wrist in the after group

	Wrist radiograph, *N* = 203	No wrist radiograph, *N* = 22	*P* value
Overall ED length of stay [median (IQR)]	1:58 (1:25–2:42)	1:24 (0:55–1:54)	0.015

*IQR* interquartile range

### Physician compliance

The physicians involved in implementing the AWR included 193 surgical residents and 209 emergency physicians. The physicians adhered to the AWR in 23 patients (36%). The main reason for not adhering to the AWR was the suspicion of an associated injury (71%). In 66%, the physicians indicated that they had the suspicion of a fracture of one of the carpal bones, in 24% of a distal ulna fracture, and in 10% they had the suspicion of other associated injuries. Of all patients who received a wrist radiograph despite the recommendation, two patients had a scaphoid fracture and one had a fracture of the triquetrum. All were treated non-operatively. No distal radius, distal ulna fractures or associated ligamentous injuries were diagnosed.

**Fig. 5:**
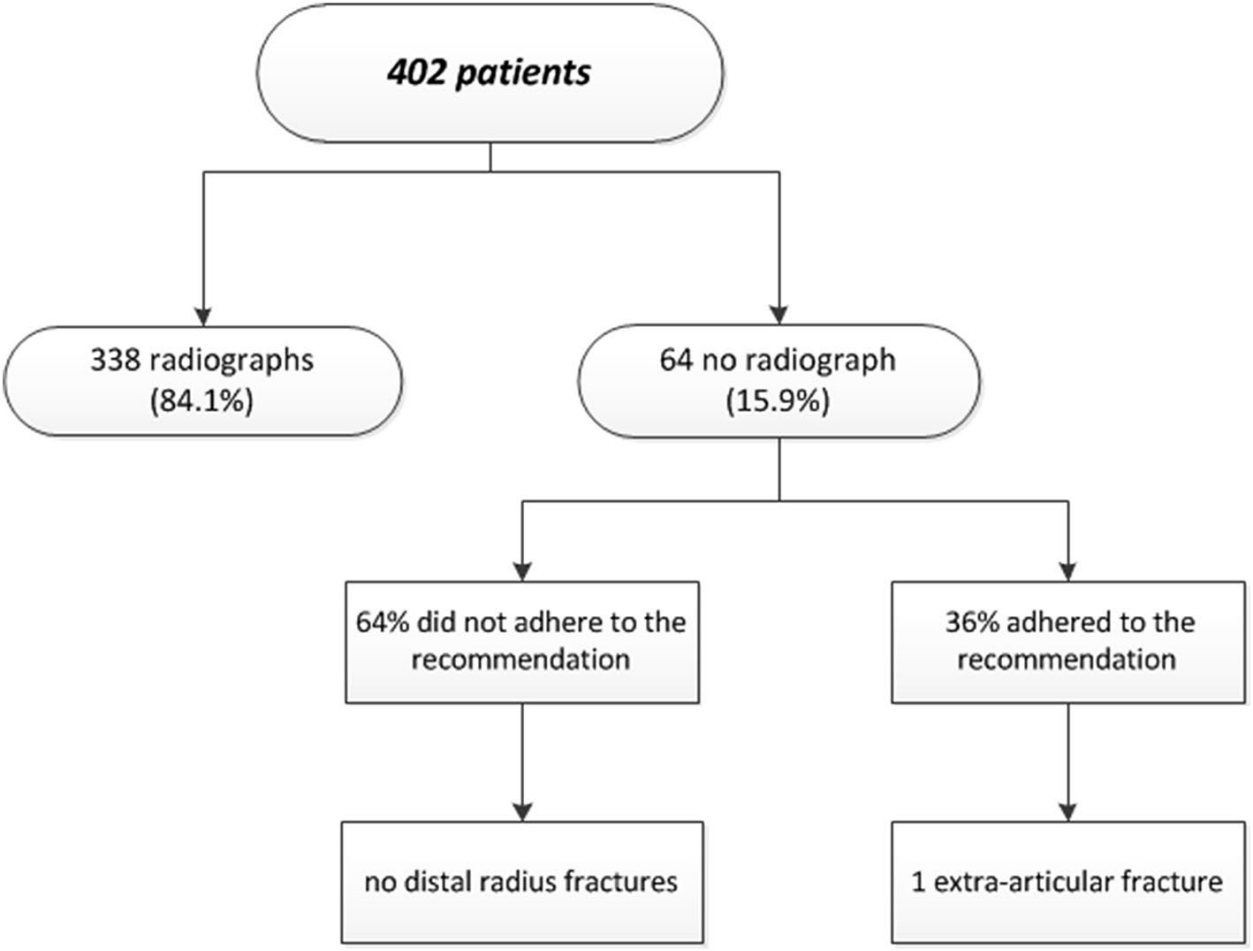
Physician compliance and missed fractures

Other reasons for physicians not adhering to the AWR were because patients insisted on a radiograph (15%), the physician disagreed with the AWR (7%), and undefined reasons (7%). There were no distal radius fractures among the patients in whom the recommendation of the AWR was not followed (Fig. 5).

Among the patients who received no wrist radiograph, eight patients received a radiograph of the hand due to the suspicion of a carpal or metacarpal fracture. Of these patients, one patients had a pisiform fracture and one patient had a fracture of the triquetrum.

### Patient satisfaction

Of the 23 patients who did not receive a radiographic examination of the wrist, 87% were satisfied with their consultation at the ED. Three patients indicated that they felt less satisfied and insecure because they did not receive a radiographic evaluation of the wrist. These three patients indicated that they would have felt more secure if a radiograph of the wrist had been made and they would have been willing to wait longer at the ED to be 100% a distal radius fracture was ruled out.

## Discussion

Implementation of the AWR resulted in a potential reduction of wrist radiographs, which was higher than the expected 14.2% based on the external validation study [3]. In addition, the sensitivity and specificity were also higher compared to the external validation study (98% and 25% versus 100% and 28%) [3]. One fracture, which was considered to be clinically irrelevant in retrospect, was missed. This fracture occurred in a 55-year-old intoxicated woman. The probability of missing a fracture can be decreased by a reliable physical examination. Therefore, we recommend not using the AWR if the patient is difficult to examine due to intoxication or distracting injuries. This same exclusion criterion is used in the NEXUS criteria for cervical spine injury [13].

With the AWR, we could potentially achieve a 15% reduction in wrist radiographs without missing any clinically relevant fractures. While a larger reduction may seem more desirable, this will also result in a lower sensitivity and, therefore, a higher percentage of missed fractures, which is undesirable. Moreover, on a national level (or even an international level) it corresponds to thousands of radiographs and, therefore, would have a large impact.

Physicians might perceive that patients are only satisfied if a fracture has been ruled out by a radiograph. In contrast, our study shows that 87% of the patients who did not receive a radiograph of the wrist were satisfied. Similar results were found after implementation of the Canadian C-Spine Rule and after introducing the Ottawa Ankle Rules [14–16]. Moreover, reassurance and medicolegal factors also affect decision making [6]. Physicians might be fearful of the medical and possible legal consequences of missing a fracture. An important aspect in managing patients without a radiograph of the wrist is counselling. During our telephone survey, patients indicated that they would not insist on a radiograph if a physician explained to them why a radiograph was unnecessary and informed them about the natural course. Moreover, counselling will take only a few minutes. While conducting and reviewing the radiograph and explaining the findings to the patient in a second interaction will take more time. This was confirmed by the 34-min reduction in time spent in the ED for non-fracture patients without a wrist radiograph. In addition, our study shows that after implementation of the AWR, selected patients can be safely sent home without a radiograph of the wrist and with instructions to contact either their general practitioner or the hospital after 7–10 days if symptoms persist.

The AWR was successfully implemented by a variety of different physicians, ranging from less experienced residents to experienced emergency physicians and surgical residents. However, the compliance with the AWR was low. Only 36% of the physicians adhered to the recommendation of the AWR, resulting in an actual reduction in wrist radiographs of 5.7%. Whereas, if all physicians would have complied, the reduction would have been 15%. The main reason for not adhering to the AWR was the suspicion of an associated injury, mainly a fracture of a carpal bone. In the 64 patients in whom no radiograph was recommended due to the AWR, seven patients had a fracture of one of the carpal bones (two scaphoid, three triquetrum and two pisiform). However, in four of these patients, additional radiographs of the hand or CT-scans were performed due to the suspicion of a carpal bone fracture by the treating physician during physical examination. One additional scaphoid fracture was diagnosed later. This patient had no clinical signs of a scaphoid fracture during physical examination, i.e. no anatomic snuffbox tenderness and no pain during axial compression of the thumb. He presented to the ED 1 week following trauma because of persistent pain. There were no distal radius fractures among the patients in which the AWR recommended not to make a wrist radiograph, and instead a radiograph was taken upon the decision of the physician.

A possible barrier of implementation of the Amsterdam Wrist Rules is that the management of a patient presenting following an acute wrist injury is not directed exclusively to the diagnosis and management of a distal radius fracture solely. Therefore, the AWR is only appropriate for those suspected of having a distal radius fracture. The AWR is not designed to replace a proper physical examination and never will replace the clinical experience of the physician, but it is a validated tool to guide physicians in the decision to request a radiograph of the wrist in case of a suspected distal radius fracture. When due to a thorough physical examination of the carpus a carpal ligamentous injury or fracture is suspected, additional specific radiographic imaging should be requested. Moreover, we are currently deriving a second clinical decision rule dedicated to detecting scaphoid fractures.

In addition, except for the telephone survey after 1 week, we did not follow-up on the patients who did not receive a wrist radiograph, and, therefore, we do not know if these patients would have developed residual complaints (e.g. due to ligamentous injuries). Patients with ligamentous injuries (e.g. S-L dissociation, TFCC lesion) are normally not diagnosed with these injuries at the ED, but present at the outpatient clinic with residual complaints. Often additional diagnostics like MRI, wrist cineradiography or even wrist arthroscopy are necessary to diagnose and treat these patients with ligamentous injuries. Nonetheless, this also applies to the patients who received a radiograph but did not have a fracture. These patients could also have associated ligamentous injuries which could arise several weeks after trauma. Therefore, it remains important to explain to patients with a negative radiograph for a distal radius fracture, that if symptoms persist they should contact a physician.

Moreover, in our cohort of patients, 25 patients (6%) were diagnosed with a clinically suspected scaphoid fracture despite negative wrist radiographs. All 25 patients were initially immobilised for 7–10 days with a cast until further diagnostics were performed. After additional diagnostics, three patients were diagnosed with a scaphoid fracture. Scaphoid fractures are often occult on plain radiographs of the wrist, and, therefore, additional diagnostics, including radiographs of the carpal bones and even a CT-scan or MRI, are necessary to detect these fractures [17–20].

Other possible barriers for implementation of a clinical decision rule are forgetting the details of the rule, its use requiring too much time, and basing the rule on flawed evidence [11]. Using a smart phone application for the AWR, we aimed to reduce these barriers. Recently, a clinical decision support tool was also successfully used to increase the adherence of the Ottawa Ankle Rules [21]. Additionally, (electronic) handheld devices with clinical decision rules, like mobile applications, have been shown to be more effective and increase adherence compared to non-handheld devices [22]. By showing that the AWR can safely be used, we expect that the adherence of physicians towards the AWR will increase in the future.

After the AWR were introduced at the end of 2015, Karaca et al. proposed the Karadeniz wrist rules [1]. The results obtained are promising, showing a 100% sensitivity and negative predictive value. Yet, the specificity is lower compared to the AWR (7% versus 28%) and the reduction in the number of radiographs taken is unknown. Moreover, the Karadeniz wrist rules have not been externally validated and implemented. Therefore, the clinical applicability has yet to be proven.

This study has some limitations. First, we chose not to randomise but used a before-and-after prospective design. The reason for this was that randomising all patients would not be feasible because wrist trauma is common and, therefore, cognitive guidelines would be learned by the physicians [10]. However, we tried to diminish selection bias by aiming to include all prospective patients with trauma of the wrist. The cohort of missed inclusions had a significantly lower distal radius fracture percentage and was significantly younger, which could imply selection bias. In contrast, the fact that there were no differences in baseline characteristics between the before-and-after group of the included patients, suggests that patients were not selected on age, fracture type or other clinical characteristics and are, therefore, comparable. Moreover, all missed inclusions received a radiograph at the ED and, therefore, we know that 34% of the patients had a fracture of the distal radius.

Second, we assessed patient satisfaction by a short telephone survey after 1 week. Although 87% of patients indicated that they were satisfied with their consultation at the ED and the fact that they did not receive a wrist radiograph, these results only represents a small sample. Moreover, there are many factors which may determine satisfaction which are not taken into account.

Third, because of the implementation of the AWR, the EDs had to change their workflow. In current practice, all patients are seen by an ED triage nurse and are either sent to the general practitioner or sent for a radiograph of the wrist even before an emergency physician or surgical resident has examined the patient. This could have incorporated an unwanted decreased compliance and led to the missed inclusions. The next step will be to implement the AWR by the ED triage nurses. This has already successfully been done for the Ottawa Ankle Rules, the Canadian C-spine Rule, and for patients with acute cardiac ischaemia [23–25]. Moreover, we estimate that the reduction in radiographs and the time spent in the ED could also result in cost savings. Therefore, we are currently undertaking a comprehensive cost analysis and budget impact analysis, taking into account direct costs such as the consultation at the ED and other health care providers and the costs of the radiographs taken. We also are including indirect costs such as time spent in the ED. Moreover, other patients at the ED could potentially benefit because physicians have more time to examine those patients. All of this could improve efficiencies at the ED, resulting in better use of resources.

The AWR is the first externally validated and implemented clinical decision rule for adult patients with wrist trauma. This implementation study shows that the AWR safely reduces the amount of wrist radiographs in selected patients and consequently the length of stay in the ED.

## Electronic supplementary material

Below is the link to the electronic supplementary material.

Supplementary material 1 (DOCX 13 kb)

Supplementary material 2 (DOCX 13 kb)
