# Supplementary material for: The Amsterdam Wrist Rules to reduce the need for radiography after a suspected distal radius fracture: an implementation study
Source: Eur J Trauma Emerg Surg. 2019 Sep 20;46(3):573–82. doi: 10.1007/s00068-019-01194-2 (PMC7280343; doi:10.1007/s00068-019-01194-2)
Supplement: Supplementary file 1 — Supplementary material 1 (DOCX 13 kb) [file 68_2019_1194_MOESM1_ESM.docx]

| Appendix 1. AWR algorithm |
| --- |
| Linear predictor^b^  0.0341* age + 1.7298 * (if swelling of distal radius present) + 1.6462 *(if visible deformation present) + 1.8117 * (if distal radius tender to palpation) + 0.4228 *(if palmar flexion is tender) + 0.6567 * (if supination is tender) – 0.2941 (if ulnar deviation is tender) + 0.5949 * (If radioulnar ballottement test is tender) - 6.0202 |
| Probability of a fracture based on final model  1/ (1+EXP(-Linear Predictor)) |
