# Supplementary material for: The Amsterdam Wrist Rules to reduce the need for radiography after a suspected distal radius fracture: an implementation study
Source: Eur J Trauma Emerg Surg. 2019 Sep 20;46(3):573–82. doi: 10.1007/s00068-019-01194-2 (PMC7280343; doi:10.1007/s00068-019-01194-2)
Supplement: Supplementary file 2 — Supplementary material 2 (DOCX 13 kb) [file 68_2019_1194_MOESM2_ESM.docx]

| **Appendix 2. Baseline characteristics of included patients versus missed inclusions** | | | |
| --- | --- | --- | --- |
|  | **Included in AWR**  **N = 402** | **Missed inclusions**  **N = 1184** | **P-value** |
| **Age** (median (IQR)) | 51 (32 – 67) | 45 (26 – 63) | <0.001* |
| **Female** (%) | 60.7 | 57.3 | 0.240 |
| **Distal radius fractures** (%) | 44 | 34 | 0.001* |
| Missed inclusions are patients who fulfilled the inclusion criteria but were not included by unknown reasons. * statistical significance; N: number; IQR: interquartile range | | |  |
